# Supplementary material for: In Vitro Conversion of Coffea spp. Somatic Embryos in SETIS™ Bioreactor System
Source: Plants (Basel). 2023 Aug 25;12(17):3055. doi: 10.3390/plants12173055 (PMC10490467; doi:10.3390/plants12173055)
Supplement: Supplementary file 1 [file plants-12-03055-s001.zip › plants-2562080-supplementary.pdf]

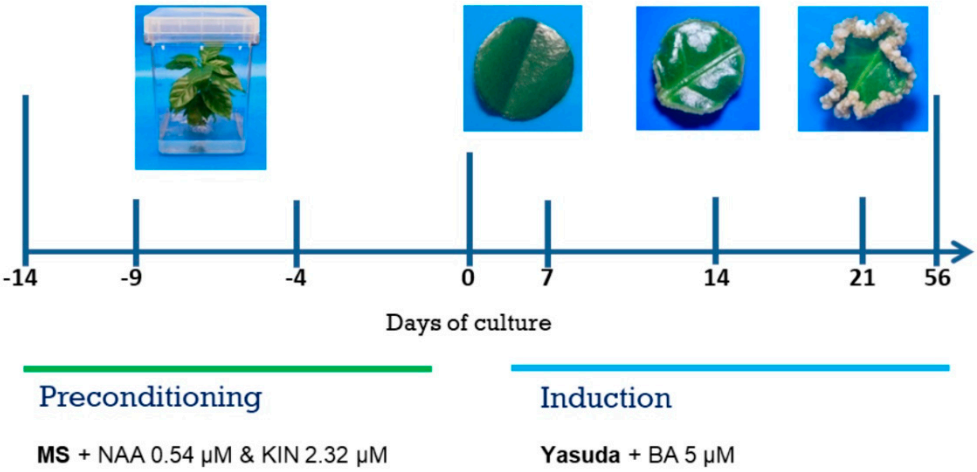

Figure S1. Graphical description of SE induction in *Coffea canephora.* First, the seedlings were preconditioned for 14 days in a semi-solid MS {Murashige, 1962 142 /id} medium supplemented with 0.54 µM NAA and 2.32 µM KIN under photoperiod conditions of 16 h light/8 h dark (150 µmol $m^{-2}s^{-1}$) at 25 ± 2 ºC. At the end of the preconditioning, the leaves were selected and cut into segments 0.8 cm in diameter with the help of a sterile punch. Five explants were placed per 250 mL flask, with 50 mL of Yasuda liquid medium with the nitrogen source modified {Yasuda, 1985 17604 /id} and supplemented with 5 µM of BA, adjusted to pH 5.8 {Quiroz-Figueroa, 2006 25818 /id}. The flasks were incubated in the dark and shaken (55 rpm) at 25 ± 2 ºC for 56 days.

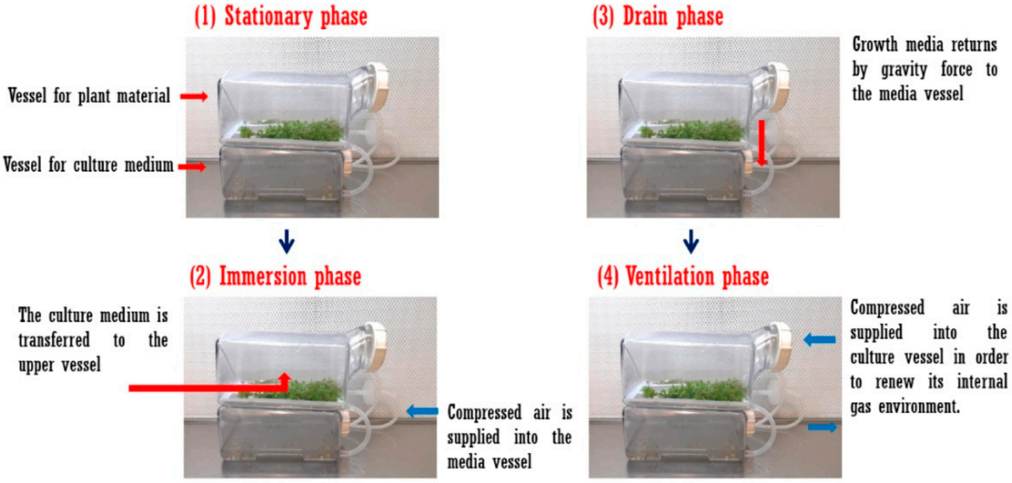

Figure S2. Description of the SETIS^{TM} bioreactor system. The SETISTM bioreactor system uses the Twin-bottles principle of two connected vessels, one for plant material and one for culture medium. The operation consists of 4 phases: (**1**) a stationary phase where no compressed air is supplied; the culture medium remains in the container and the plant material within a gaseous environment, (**2**) an immersion phase where compressed air is supplied to the culture medium container to transfer it to the plant material container, (**3**) a drainage phase in which the culture medium returns by force of gravity to the medium container, (**4**) a ventilation phase where compressed air is supplied to the culture vessel to renew its gaseous environment (https://setis-systems.be/products/setis-bioreactor).

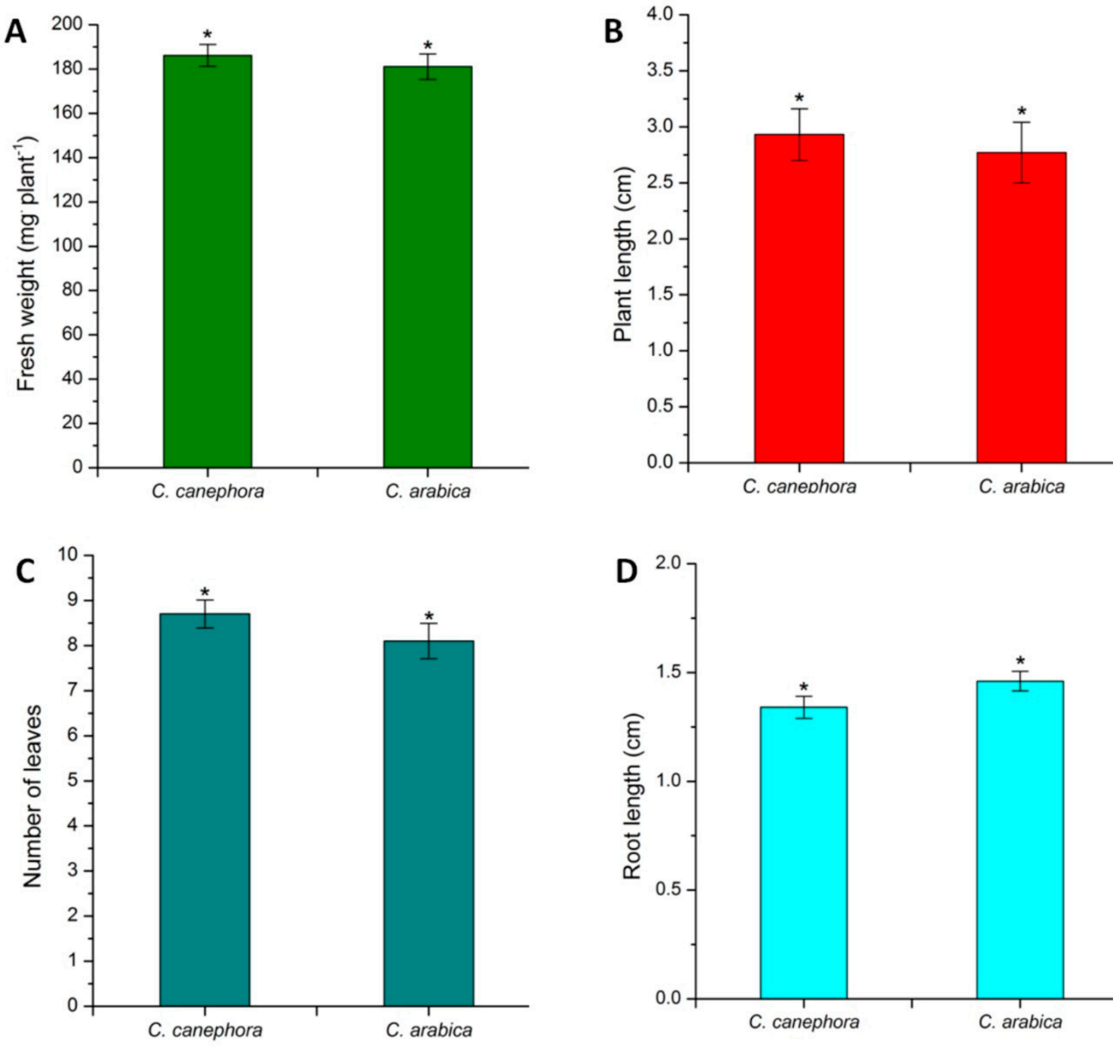

Figure S3. Growth evaluation of *Coffea* spp. plants after ten weeks of culture in bioreactors. (**A**) Fresh weight, (**B**) Plant length, (**C**) Number of leaves, (**D**) Root length. Error bars represent standard error. Means followed by an asterisk are not significantly different (T-test $P \leq 0.05$).

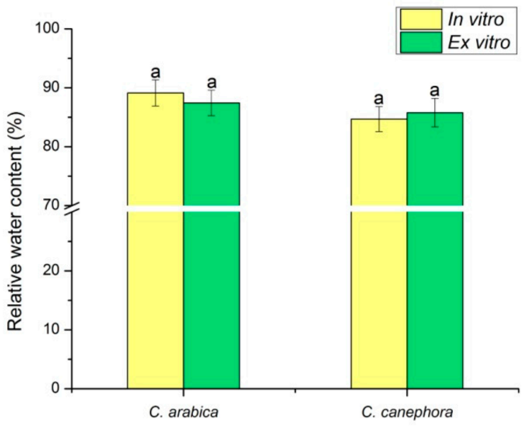

Figure S4. Relative water content (RWC) of *C. arabica* and *C. canephora* plants under *in vitro* and *ex vitro* conditions. Error bars represent standard error. Means followed by the same letter are not significantly different (Tukey test $P \leq 0.05$).

Table S1. Descriptive statistics and analysis of variance of the substrate proportion tests for the acclimatization of *Coffea* spp. plants.

Descriptive satistics			
Substrate	Mean	Standard Deviation	SE of Mean
GS:A (1:1)	40	6.67	3.85093
GS:PM (1:1)	57.77667	3.85093	2.22333
PM:A (1:1)	71.11	3.84515	2.22
GS:PM:A (2:1:1)	64.44667	3.85093	2.22333
GS:PM:A (1:1:1)	82.22333	3.85093	2.22333
GS:PM:A (1:1:0.5)	91.11	3.84515	2.22

ANOVA					
	DF	Sum of Squares	Mean Square	F Value	Prob>F
Model	5	4940.68151	988.1363	50.01188	1.27E-07
Error	12	237.0964	19.75803		
Total	17	5177.77791			

Table S2. Descriptive statistics and t-student test of the growth related variables evaluated in regenerated plants of *Coffea* spp.

Descriptive statistics and t-student test of the fresh weight from regenerated plantlets.

DESCRIPTIVE STATISTICS				
	N	Mean	SD	SEM
C. canephora	15	186.16	7.41531	1.91463
C. arabica	9	181.06667	2.37908	0.79303

t-TEST STATISTICS			
	t Statistic	DF	Prob>|t|
Equal Variance Assumed	1.98459	22	0.0598
Equal Variance NOT Assumed	2.45774	18.27439	0.02418

Descriptive statistics and t-student test of the length from regenerated plantlets.

DESCRIPTIVE STATISTICS				
	N	Mean	SD	SEM
C. canephora	15	2.93333	0.2743	0.07082
C. arabica	9	2.77778	0.22791	0.07597

t-TEST STATISTICS			
	t Statistic	DF	Prob>|t|
Equal Variance Assumed	1.42779	22	0.1674
Equal Variance NOT Assumed	1.4977	19.52184	0.1502

Descriptive statistics and t-student test of the leaves number from regenerated plantlets.

DESCRIPTIVE STATISTICS				
	N	Mean	SD	SEM
C. canephora	15	8.66667	1.23443	0.31873
C. arabica	9	8	1.41421	0.4714

t-TEST STATISTICS			
	t Statistic	DF	Prob>|t|
Equal Variance Assumed	1.21376	22	0.23771
Equal Variance NOT Assumed	1.17156	15.17408	0.25944

Descriptive statistics and t-student test of the root length from regenerated plantlets.

DESCRIPTIVE STATISTICS				
	N	Mean	SD	SEM
C. canephora	15	1.34	0.45638	0.11784
C. arabica	9	1.46667	0.31623	0.10541

t-TEST STATISTICS			
	t Statistic	DF	Prob>|t|
Equal Variance Assumed	-0.73097	22	0.47251
Equal Variance NOT Assumed	-0.80116	21.39541	0.43185
